# Characteristics and Outcomes of Adults With Congenital Heart Disease in the Cardiac Intensive Care Unit

**DOI:** 10.1016/j.jacadv.2024.101077

**Published:** 2024-07-22

**Authors:** Ryan R. Keane, Anthony P. Carnicelli, Daniel B. Loriaux, Payton Kendsersky, Richard A. Krasuski, Kelly M. Brown, Kelly Arps, Vivian Baird-Zars, Jeffrey A. Dixson, Emily Echols, Christopher B. Granger, Robert W. Harrison, Michael Kontos, L. Kristin Newby, Jeong-Gun Park, Kevin S. Shah, Bradley W. Ternus, Sean Van Diepen, Jason N. Katz, David A. Morrow

**Affiliations:** a Department of Cardiovascular Medicine, Cleveland Clinic Foundation, Cleveland, Ohio, USA; b Division of Cardiology, Department of Medicine, Medical University of South Carolina, Charleston, South Carolina, USA; c Division of Cardiology, Department of Medicine, Duke University Hospital, Durham, North Carolina, USA; d TIMI Study Group, Cardiovascular Division, Brigham and Women’s Hospital, Harvard Medical School, Boston, Massachusetts, USA; e Division of Cardiology, Department of Medicine, Virginia Commonwealth University School of Medicine, Richmond, Virginia, USA; f Division of Cardiology, Department of Medicine, University of Utah, Salt Lake City, Utah, USA; g Division of Cardiology, Department of Medicine, Mayo Clinic, Rochester, Minnesota, USA; h Department of Critical Care Medicine and Division of Cardiology, Department of Medicine, University of Alberta, Alberta, Canada

**Keywords:** adult congenital heart disease, ACHD, cardiac intensive care unit, CICU

## Abstract

**Background:**

Little is known regarding the characteristics, treatment patterns, and outcomes in patients with adult congenital heart disease (ACHD) admitted to cardiac intensive care units (CICUs).

**Objectives:**

The authors sought to better define the contemporary epidemiology, treatment patterns, and outcomes of ACHD admissions in the CICU.

**Methods:**

The Critical Care Cardiology Trials Network is a multicenter network of CICUs in North America. Participating centers contributed prospective data from consecutive admissions during 2-month annual snapshots from 2017 to 2022. We analyzed characteristics and outcomes of admissions with ACHD compared with those without ACHD. Multivariable logistic regression was used to assess mortality in ACHD vs non-ACHD admissions.

**Results:**

Of 23,299 CICU admissions across 42 sites, there were 441 (1.9%) ACHD admissions. Shunt lesions were most common (46.1%), followed by right-sided lesions (29.5%) and complex lesions (28.7%). ACHD admissions were younger (median age 46 vs 67 years) than non-ACHD admissions. ACHD admissions were more commonly for heart failure (21.3% vs 15.7%, *P* < 0.001), general medical problems (15.6% vs 6.0%, *P* < 0.001), and atrial arrhythmias (8.6% vs 4.9%, *P* < 0.001). ACHD admissions had a higher median presenting Sequential Organ Failure Assessment score (5.0 vs 3.0, *P* < 0.001). Total hospital stay was longer for ACHD admissions (8.2 vs 5.9 days, *P* < 0.01), though in-hospital mortality was not different (12.7% vs 13.6%; age- and sex-adjusted OR: 1.19 [95% CI: 0.89-1.59], *P* = 0.239).

**Conclusions:**

This study illustrates the unique aspects of the ACHD CICU admission. Further investigation into the best approach to manage specific ACHD-related CICU admissions, such as cardiogenic shock and acute respiratory failure, is warranted.

The population of patients with adult congenital heart disease (ACHD) is growing rapidly.^1^^,^^2^ As this population ages, so does their burden of both cardiovascular and noncardiovascular comorbidities and health care utilization.^3^, ^4^, ^5^ Compared to the general population, patients with ACHD have an increased likelihood of requiring admission to an intensive care unit (ICU) and have a higher mortality for many common noncardiovascular conditions requiring hospitalization.^6^ ACHD patients are highly diverse with unique anatomy, hemodynamics, and extracardiac physiology that requires special consideration, particularly in the setting of critical illness.^7^^,^^8^ Where available, ACHD patients hospitalized with critical illness are typically admitted to a specialized cardiac intensive care unit (CICU). There is a paucity of data describing the epidemiology, patient characteristics, and resource utilization in critically ill patients with ACHD.

We used a large prospective North American registry of admissions to CICUs to describe the baseline characteristics, treatment patterns, and outcomes in ACHD admissions to the CICU.

## Methods

### Critical care cardiology trials network registry

The Critical Care Cardiology Trials Network Registry (CCCTN) is an investigator-initiated, collaborative network of American Heart Association level 1 CICUs in the United States and Canada. Details of the inception, conduct, and methods of the CCCTN registry have been reported previously.^9^ Scientific oversight of CCCTN is conducted by its academic executive and steering committees and is coordinated by the TIMI Study Group, Brigham and Women’s Hospital, Boston, Massachusetts. All participating investigators and study coordinators undergo central CCCTN training on data collection and the central coordinating center reviews all cases for consistency. Participating centers contributed at least annual 2-month data “snapshots” of consecutive medical admissions to the CICU from 2017 through 2022. Only the first CICU admission during a hospitalization was collected; readmission to the CICU was recorded, but no further data for the subsequent CICU stay during the same hospitalization was collected. A patient admitted in two separate snapshots (eg, 2017 and 2019) would be recorded twice as these were two separate CICU admissions. A detailed description of the data elements recorded for the CCCTN registry has been reported previously.^10^ The CCCTN registry protocol and waiver of informed consent were approved by the Institutional Review Board at the data coordinating center (Brigham and Women’s Hospital) and each participating center. No protected health information is collected in the CCCTN registry.

### Congenital heart disease study population

The CCCTN registry collects information regarding baseline characteristics, primary admission diagnosis, indications for CICU admission, clinical CICU course, and hospital outcomes as well as the presence of cardiac arrest or shock. All data are obtained by chart review.

For this study, we analyzed CICU admissions with a history of ACHD as documented on the case report form. For the first two annual data collection periods (2017 and 2018), ACHD was documented as present or absent, without collecting further details describing specific lesion types. Starting in 2019, admissions with ACHD were further classified in the case report form into specific subcategories based on lesion type (shunt lesions, left-sided lesions, right-sided lesions, coronary anomalies, and complex lesions), as defined in Table 1. Admissions could be classified as having multiple types of lesions, and thus lesions are not mutually exclusive. Although we used a framework for classification supported by professional society guidelines,^11^ the registry does not capture further subclassifications and therefore does not support further detailing of specific lesions. Data described include primary and secondary admission diagnosis, demographics, comorbidities, CICU resource utilization, CICU mortality, in-hospital mortality, and length of stay. Admission labs within the first 24 hours of admission to the CICU were also captured. A Sequential Organ Failure Assessment (SOFA) was calculated based on admission labs and vitals (PaO_2_, FiO_2_, mean arterial pressure/vasopressor requirement, Glasgow Coma Scale, platelet count, bilirubin, and creatinine).^12^

**Table 1:** Congenital Heart Lesion Categories

Congenital Lesion	Types of Lesions
Shunt lesion	ASD, PAPVC, VSD, AV septal defect, PDA
Right-sided lesions	Tetralogy of Fallot, pulmonic stenosis/regurgitation, Ebstein’s anomaly
Complex lesions	TGA, single ventricle physiology, Fontan circulation, truncus arteriosus, hypoplastic ventricles, Eisenmenger syndrome/severe PAH
Left-sided obstructive lesion	Bicuspid aortic valve, coarctation of the aorta, congenital mitral stenosis, subvalvular/supravalvular aortic stenosis, aortopathies,
Coronary anomalies	N/A
Other	Not otherwise classified

ASD = atrial septal defect; AV = atrioventricular; PAH = pulmonary arterial hypertension; PAPVC = partial anomalous pulmonary venous connection; PDA = patent ductus arteriosus; TGA = transposition of great arteries; VSD = ventricular septal defect.

### Statistical analysis

Admissions with ACHD were contrasted for descriptive purposes to those without ACHD. Continuous variables were compared using the Wilcoxon rank-sum test and categorical variables were compared using either the chi-squared or Fisher’s exact test, as appropriate. All tests were 2-sided. No adjustments were made for multiple comparisons, and interval estimates should be interpreted with caution given lack of control for multiplicity.

Multivariable logistic regression was performed to assess mortality (CICU and in-hospital) in ACHD vs non-ACHD admissions adjusting for age, sex, and SOFA score. All analyses were performed using SAS, version 9.4 (SAS Institute).

## Results

### Baseline characteristics and admission diagnosis

During the study period, 23,299 admissions to participating CICUs were recorded, among which 441 (1.9%) admissions had a documented history of ACHD. Baseline characteristics are listed in Table 2. Compared to admissions without ACHD, those with ACHD were younger (median age 46 vs 67 years, *P* < 0.001) and more commonly female (44.2% vs 36.5%, *P* < 0.001). Severe valvular heart disease (27.0% vs 14.5%, *P* < 0.001), atrial fibrillation (40.8% vs 25.2%, *P* < 0.001), significant liver disease (12.0% vs 2.8%, *P* < 0.001), heart failure (44.2% vs 36.3%, *P* < 0.001), and pulmonary hypertension (22.7% vs 5.3%, *P* < 0.001) were all more prevalent among admissions with ACHD. Conversely, admissions with ACHD less frequently had diabetes (15.4% vs 34.9%, *P* < 0.001), hypertension (27.0% vs 64.8%, *P* < 0.001), and coronary artery disease (11.3% vs 36.3%, *P* < 0.001). There were 254 admissions with specific congenital subcategorization available (Table 3). The three most common categories were shunt lesions (46.1%), right-sided lesions (29.5%), and complex lesions (28.7%).

**Table 2:** Demographics and Baseline History in Patients With and Without Adult Congenital Heart Disease Admitted to the Cardiac Intensive Care Unit

	Admissions With ACHD (n = 441)	Admissions Without ACHD (n = 22,858)	*P* Value
Demographics			
Age, y	46 (33-60)	67 (57-76)	<0.001
Female	195 (44.2%)	8,349 (36.5%)	<0.001
Black	46 (12.5%)	3,906 (21.2%)	<0.001
Hispanic/Latino	37 (8.4%)	1,658 (7.3%)	0.048
BMI, kg/m^2^	26.2 (22.5-31.5)	27.7 (24.0-32.5)	<0.001
Medical history			
Current smoker	32 (7.3%)	3,946 (17.3%)	<0.001
Pulmonary disease	70 (15.9%)	3,394 (14.8%)	0.549
Liver disease	53 (12.0%)	638 (2.8%)	<0.001
Chronic kidney disease	87 (19.7%)	5,447 (23.8%)	0.045
Diabetes mellitus	68 (15.4%)	7,970 (34.9%)	<0.001
Hypertension	119 (27.0%)	14,819 (64.8%)	<0.001
Active cancer	9 (2.0%)	1,594 (7.0%)	<0.001
Cardiovascular history			
Coronary artery disease	50 (11.3%)	8,302 (36.3%)	<0.001
Heart failure	195 (44.2%)	8,293 (36.3%)	<0.001
Systemic EF <50%	111/195 (62.4%)	5,832/8,293 (72.8%)	0.002
Atrial fibrillation	180 (40.8%)	5,750 (25.2%)	<0.001
Ventricular arrhythmia	50 (11.3%)	1,413 (6.2%)	<0.001
Significant valvular disease	119 (27.0%)	3,309 (14.5%)	<0.001
Pulmonary hypertension	100 (22.7%)	1,220 (5.3%)	<0.001

Values are median (25th-75th percentile) or n (%).

ACHD = adult congenital heart disease; BMI = body mass index; EF = ejection fraction.

**Table 3:** Number of Admissions in Each of the Congenital Heart Lesion Categories (N = 254)

Shunt lesion	117 (46.1%)
Right-sided lesions	75 (29.5%)
Complex lesions	73 (28.7%)
Left-sided obstructive lesion	38 (15.0%)
Coronary anomalies	9 (3.5%)
Other	4 (1.6%)

Values are n (%). Specific lesion classification was available for 254/441 (58%) of the total ACHD population.

CICU admission labs and worst lab values are listed in Table 4. Admissions with ACHD had a higher median SOFA score compared to those without ACHD (5 [IQR: 2-8] vs 3 [IQR: 1-6], *P* < 0.001) and more frequently had a SOFA score >8 (26.3% vs 19.6%, *P* < 0.001). Admissions with ACHD also less frequently had a normal total bilirubin (defined as <1.2 mg/dL) compared to admissions without ACHD (58.3% vs 75.4%, *P* < 0.001).

**Table 4:** Admissions and Worst Laboratory Values Stratified by Admissions With and Without Adult Congenital Heart Disease

	Admissions With ACHD (n = 441)	Admissions Without ACHD (n = 22,858)	*P* Value
Admission labs			
Lactate (mmol/L)	1.6 (1.1-2.4)	1.8 (1.2-3.2)	<0.001
Lactate ≥4 mmol/L	34 (11.4%)	2,580 (18.6%)	0.001
Arterial pH	7.4 (7.3-7.4)	7.4 (7.3-7.4)	0.043
pH <7.2 (%)	2 (2.7%)	406 (12.2%)	0.013
eGFR (ml/min/1.73 m^2^)	70.6 (42.3-98.4)	62.2 (37.9-85.0)	<0.001
Platelets (K/μL)	180 (135-236)	211 (161-268)	<0.001
SOFA score	5.0 (2.0-8.0)	3.0 (1.0-6.0)	<0.001
SOFA ≥8	116 (26.3%)	4,478 (19.6%)	<0.001
Worst laboratory values^a^			
Lactate (mmol/L)	1.9 (1.2-3.5)	2.1 (1.3-3.9)	0.018
Arterial pH	7.4 (7.3-7.4)	7.3 (7.3-7.4)	0.157
eGFR (ml/min/1.73 m^2^)	60.1 (32.6-85.3)	54.3 (29.9-77.4)	<0.001
Platelets (K/μL)	149 (105-196)	176 (127-229)	<0.001
ALT (U/L)			
<200 U/L	336 (91.3%)	16,710 (88.9%)	0.534
200-799 U/L	17 (4.6%)	1,309 (7.0%)	
800-1999 U/L	9 (2.5%)	460 (2.4%)	
>2000 U/L	6 (1.6%)	327 (1.7%)	
Total bilirubin			
<1.2 mg/dL	214 (58.3%)	14,073 (75.4%)	<0.001
1.2-1.9 mg/dL	71 (19.3%)	2,618 (14.0%)	
2.0-5.9 mg/dL	69 (18.8%)	1,736 (9.3%)	
6.0-11.9 mg/dL	7 (1.9%)	160 (0.9%)	
≥12.0 mg/dL	6 (1.6%)	69 (0.4%)	

Values are median (25th-75th percentile) or n (%).

ACHD = adult congenital heart disease; ALT = alanine transferase; eGFR = estimated glomerular filtration rate; SOFA = Sequential Organ Failure Assessment.

a Worst lab value within 24 h of admission.

**Figure 1:**
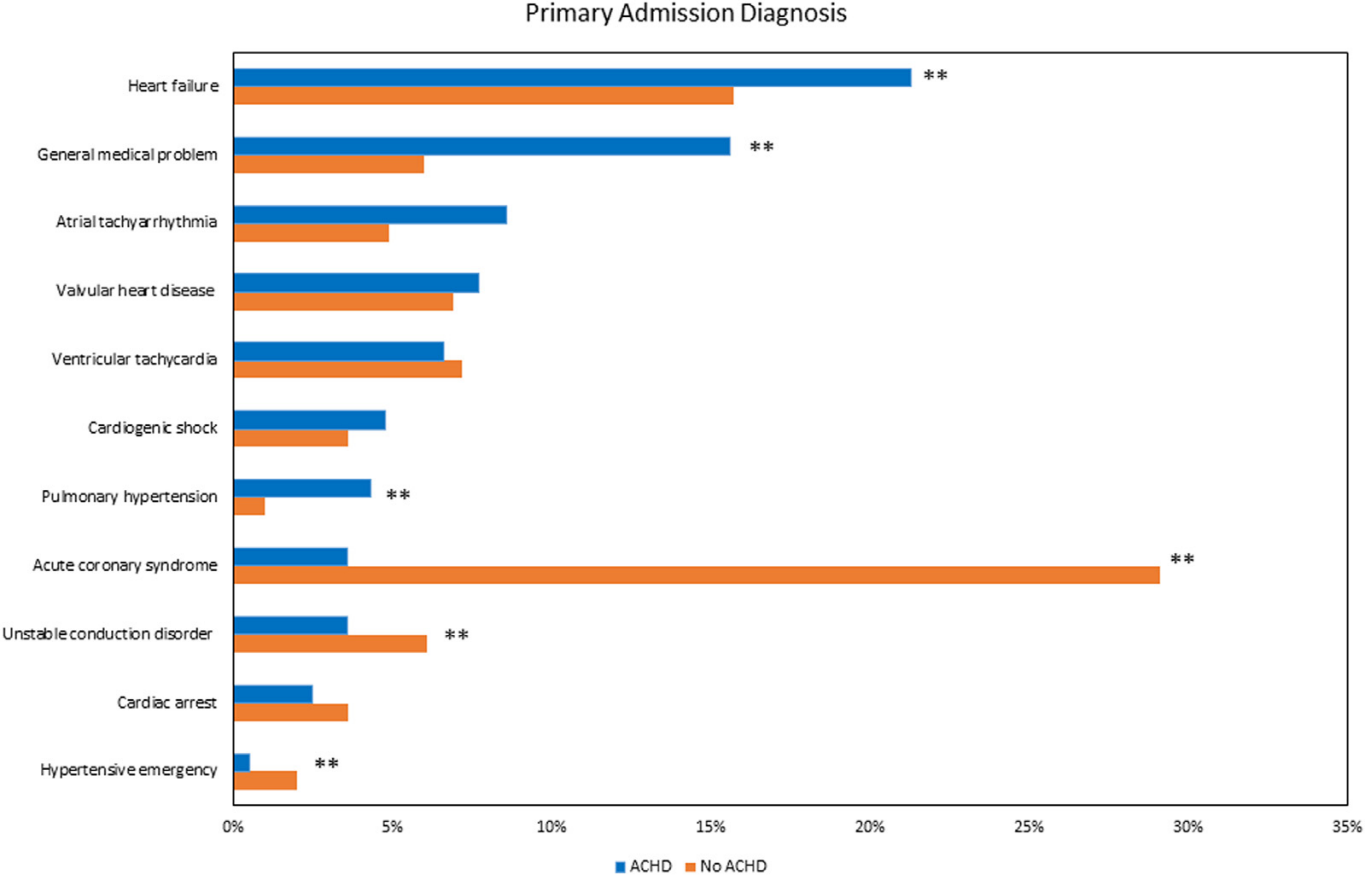
**Primary Admission Diagnosis Stratified by Admissions With and Without Adult Congenital Heart Disease** ∗∗*P* < 0.05. ACHD = adult congenital heart disease.

CICU admission diagnoses are shown in Figure 1. Compared to those without ACHD, admissions with ACHD were more commonly for heart failure (21.3% vs 15.7%, *P* < 0.001), general medical problems (15.6% vs 6.0%, *P* < 0.001), atrial tachyarrhythmias (8.6% vs 4.9%, *P* < 0.001), and management of pulmonary hypertension (4.3% vs 1.0%, *P* < 0.001). Admissions with ACHD were less commonly for acute coronary syndromes (3.6% vs 29.1%, *P* < 0.001), hypertensive emergency (0.5% vs 2.0%, *P* = 0.021), and unstable conduction disorders (3.6% vs 6.1%, *P* = 0.030).

### CICU resource utilization

**Figure 2:**
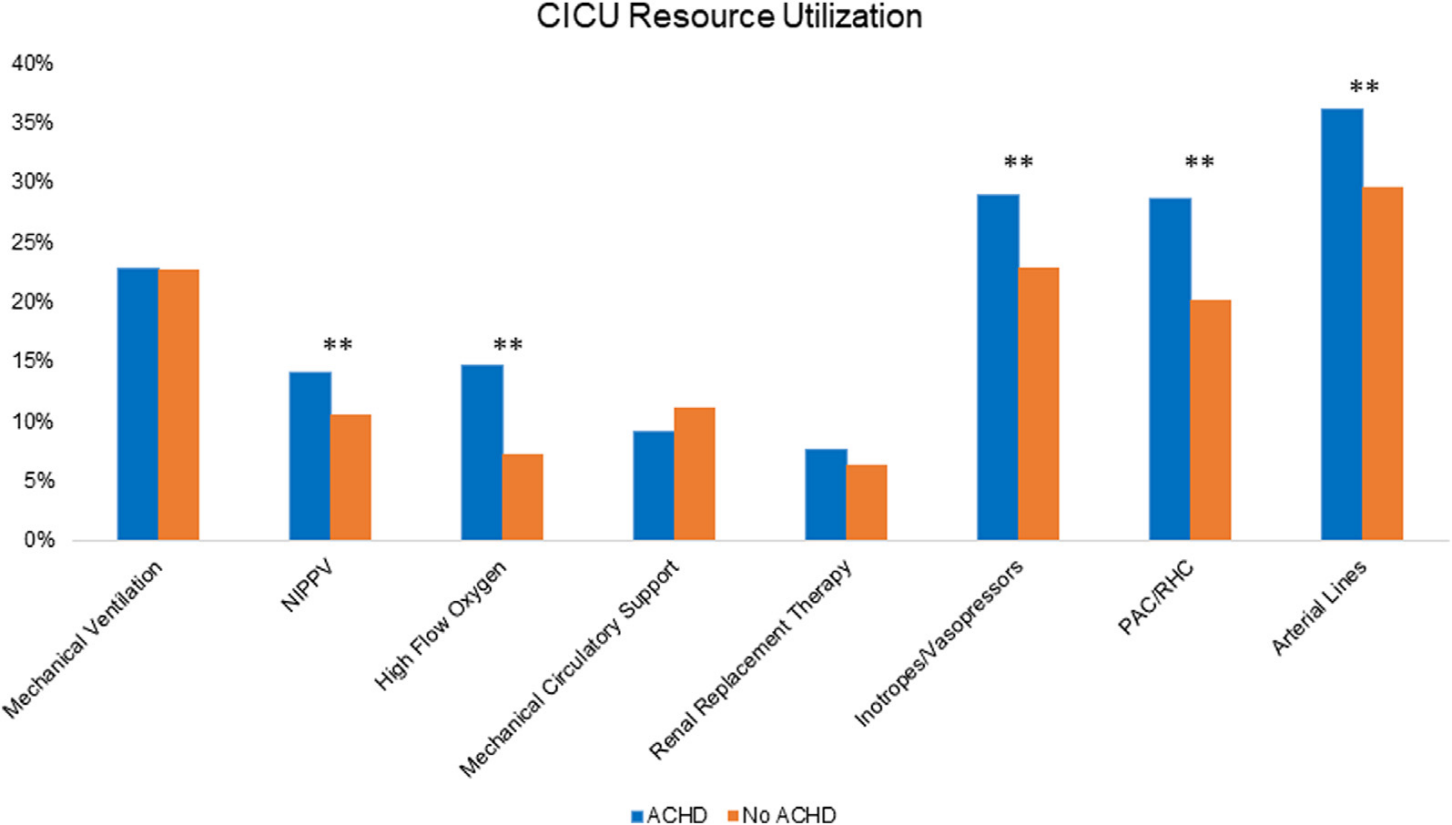
**Cardiac Intensive Care Unit Resource Utilization Stratified by Admissions With and Without Adult Congenital Heart Disease** ∗∗*P* < 0.05. ACHD = adult congenital heart disease; CICU = cardiac intensive care unit; NIPPV = noninvasive positive pressure ventilation; PAC = pulmonary artery catheter; RHC = right heart catheterization.

CICU resource utilization patterns stratified by the presence or absence of ACHD are shown in Figure 2. Admissions with ACHD more frequently received high flow oxygen (14.7% vs 7.2%, *P* < 0.001), noninvasive positive pressure ventilation (14.1% vs 10.6%, *P* = 0.019), vasopressors (30.8% vs 24.1%, *P* = 0.0017), arterial line monitoring (36.1% vs 29.7%, *P* = 0.004), and pulmonary artery catheterization (28.6% vs 20.1%, *P* < 0.001). There were no significant differences in the use of mechanical ventilation (23.4% vs 22.8%, *P* = 0.778), mechanical circulatory support (MCS) (9.1% vs 11.2%, *P* = 0.161), or renal replacement therapy (7.7% vs 6.4%, *P* = 0.280) between admissions with and without ACHD.

### Length of stay and CICU mortality

**Figure 3:**
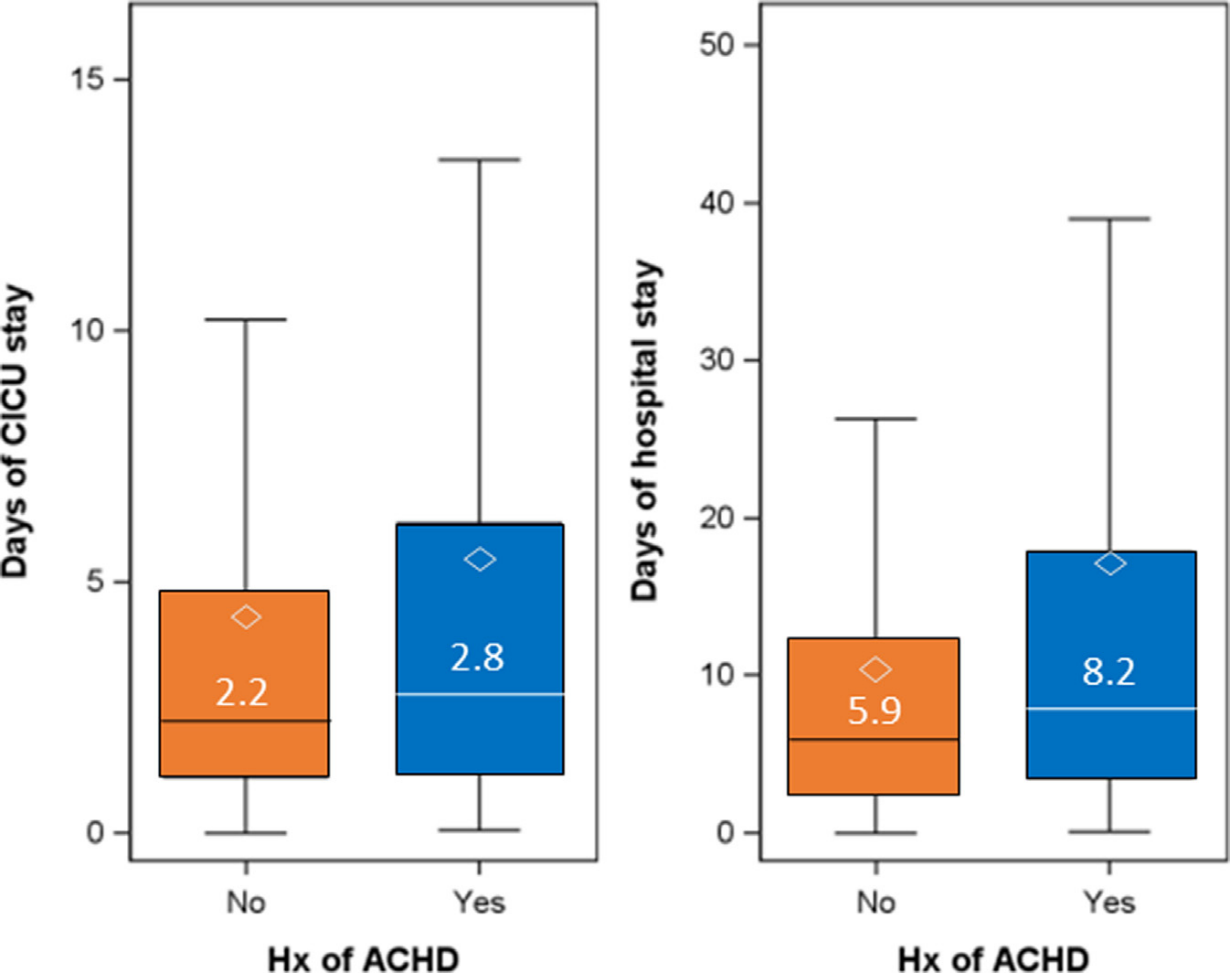
**Cardiac Intensive Care Unit and Hospital Length of Stay Stratified Admissions With and Without Adult Congenital Heart Disease** Median number of days reported in box plot. Diamond denotes the mean. ∗∗*P* < 0.05. ACHD = adult congenital heart disease; CICU = cardiac intensive care unit; Hx = history.

Median length of stays were longer for ACHD admissions compared to non-ACHD admissions in both the CICU (difference 0.58 days [95% CI: 0.31-0.85]; *P* = 0.0006) and in the hospital (difference 2.27 [95% CI: 1.0-3.5]; *P* < 0.0001) (Figure 3). CICU mortality (Table 5) was 7.5% for admissions with ACHD and 9.9% for admissions without ACHD (unadjusted OR: 0.74 [95% CI: 0.52-1.06], *P* = 0.098). In-hospital mortality was 12.7% for admissions with ACHD and 13.6% for admissions without ACHD (unadjusted OR: 0.92, [95% CI: 0.70-1.22], *P* = 0.573). Moreover, adjusting for age and sex, there remained no significant difference in adjusted CICU or in-hospital mortality (Table 5). Accounting for baseline severity of organ dysfunction by adjusting additionally for SOFA score, the odds of death in the CICU was lower for ACHD vs non-ACHD admissions (OR: 0.65 [95% CI: 0.43-0.98], *P* = 0.037), with a concordant trend in hospital mortality (OR: 0.85 [95% CI: 0.61-1.18], *P* = 0.326).

**Table 5:** Cardiac Intensive Care Unit and Hospital Mortality in Patients With and Without Adult Congenital Heart Disease

	Admissions With ACHD (n = 441)	Admissions Without ACHD (n = 22,858)	*P* Value
Mortality			
CICU mortality	33 (7.5%)	2,254 (9.9%)	0.096
In-hospital mortality	56 (12.7%)	3,115 (13.6%)	0.573
Multivariate analysis			
CICU mortality			
Unadjusted	0.74 (0.52-1.06)		0.098
Adjusted for age and sex	0.95 (0.66-1.37)		0.785
Adjusted for age, sex, and SOFA score	0.65 (0.43-0.98)		0.038
In-hospital mortality			
Unadjusted	0.92 (0.69-1.22)		0.573
Adjusted for age and sex	1.19 (0.891-1.59)		0.239
Adjusted age, sex, and SOFA score	0.85 (0.61-1.18)		0.326

Values are n (%) or OR (95% CI).

ACHD = adult congenital heart disease; CICU = cardiac intensive care unit; SOFA = sequential organ failure score.

The diagnoses with the highest mortality among admissions with ACHD were cardiogenic shock (38.1%), heart failure (18.1%), and general medical problems (14.5%) (Table 6). The majority of both ACHD and non-ACHD admissions were discharged to home or with home health services (70.6% vs 67.2%, *P* = 0.403). Readmission to the CICU was similar between admissions with and without ACHD (5.7% vs 4.3%, *P* = 0.157).

**Table 6:** Mortality Rates Stratified by Primary Admission Diagnosis in Adult Congenital Heart Disease Admissions

Admission Diagnosis	Admissions With ACHD	Admissions Without ACHD	*P* Value
General medicine problem	10/69 (14.5%)	260/1,363 (19.1%)	0.342
Atrial tachyarrhythmia	0/38 (0.0%)	81/1,112 (7.3%)	0.084
Ventricular arrhythmia	1/29 (3.4%)	262/1,651 (15.9%)	0.068
Cardiogenic shock	8/21 (38.1%)	246/826 (29.8%)	0.412
Heart failure	17/94 (18.1%)	566/3,581 (15.8%)	0.550
Pulmonary hypertension	2/19 (10.5%)	51/218 (23.4%)	0.197
Valvular heart disease	2/34 (5.9%)	189/1,586 (11.9%)	0.280

Values are n/N (%).

ACHD = adult congenital heart disease.

## Discussion

We analyzed data from the CCCTN registry to describe admissions to contemporary CICUs with ACHD (Central Illustration). Admissions with ACHD accounted for approximately 2% of all CICU admissions. Shunt lesions such as atrial septal defect represented nearly half of the analyzed population, followed closely by right-sided lesions such as tetralogy of Fallot, and then complex lesions such as single ventricle physiology and/or Fontan circulation. Admissions with ACHD were on average 20 years younger and had a unique profile of admissions diagnoses, namely more liver disease, atrial arrhythmias, and pulmonary hypertension than non-ACHD admissions. Admissions with ACHD also had a higher SOFA score and more frequently utilized advanced respiratory therapies, vasopressors, and invasive hemodynamics. Finally, ACHD admissions had longer lengths of stay in the CICU and in the hospital, but despite their substantially younger age, had similar rates of CICU readmission and in-hospital mortality. These quantitative data fill a gap in the epidemiology of this important growing population.

**Central Illustration:**
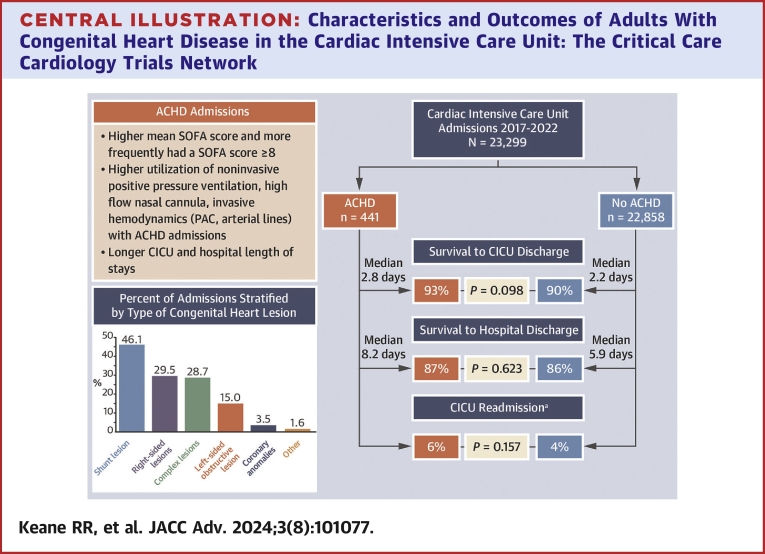
**Characteristics and Outcomes of Adults With Congenital Heart Disease in the Cardiac Intensive Care Unit: The Critical Care Cardiology Trials Network** Readmission was evaluated for index hospitalization only. ACHD = adult congenital heart disease; CICU = cardiac intensive care unit; PAC = pulmonary artery catheter; SOFA = Sequential Organ Function Score.

### Clinical presentation of patients with ACHD requiring critical care

ACHD patients are known to have increased rates of atrial arrhythmias, pulmonary hypertension, and liver disease.^4^^,^^13^, ^14^, ^15^ Our study quantifies extracardiac comorbidities as another key driver of their CICU admission, as “general medical problems” (ie, liver and renal failure, hypoxic respiratory failure, sepsis) were the second most common admission diagnosis for ACHD admissions. Mortality was not uniform across admission diagnoses; while no ACHD admissions primarily for atrial arrhythmia died, admissions for general medical problems contributed nearly 20% of the total deaths among ACHD admissions. These findings emphasize the need for clinical expertise and training in both cardiac and extracardiac complications of ACHD in the CICU setting.

Heart failure was the leading cause of ACHD admission to the CICU in our study, accounting for over 20% of all ACHD admissions. Heart failure was also the greatest contributor to deaths among ACHD admissions, accounting for 30% of deaths. This finding is consistent with previous studies in which ACHD patients admitted for heart failure had prolonged lengths of stay and higher in-hospital and 1-year mortalities compared to ACHD patients without heart failure.^16^^,^^17^ Heart failure is a heterogenous condition in ACHD and is often difficult to accurately capture solely with a left ventricular ejection fraction-focused classification, particularly in patients with Fontan or single ventricle physiology, systemic right ventricles, or severe pulmonary hypertension and right ventricular failure. This aspect can make it challenging to identify and treat such patients, particularly in centers without sufficient experience in advanced therapeutic options for ACHD. However, the rapid identification and escalation of treatment to advanced therapies for ACHD may be instrumental to prevent morbidity and mortality in this patient population. Despite the high proportion of admissions for heart failure in the ACHD group and higher utilization of vasopressors among ACHD admissions, admissions for cardiogenic shock were similar between both ACHD and non-ACHD admissions. This contrast may be in part due to an under recognition of low output states in the ACHD population given their complex physiology and prevalent cyanosis. Previous studies have found ACHD patients less frequently receive both temporary and durable MCS, likely due to more complex anatomy and more comorbid conditions.^18^^,^^19^ In our registry, MCS utilization was ∼10% for both ACHD and non-ACHD admissions, which may represent a paradigm shift toward wider use of temporary MCS in the ACHD population, although our overall sample was small and the specialized centers in our study may be more willing to adapt new technologies to this complex patient cohort.

### Critical care for patients with ACHD

Previous studies have highlighted increased ICU admissions and overall health care spending in the ACHD population relative to the general population.^5^^,^^6^ Our study found that ACHD admissions to CICUs specifically had higher utilization of certain ICU resources, namely noninvasive positive pressure ventilation, high flow nasal cannula, and invasive hemodynamics (pulmonary artery catheters and arterial lines). The emphasis on noninvasive oxygen support may be in part due to the chronic hypoxemia observed in many patients with ACHD, particularly with shunt or single ventricle physiology. The high prevalence of restrictive lung disease in Fontan and Tetralogy of Fallot patients may also contribute to the greater oxygen therapy utilization.^20^ Our study also found that admissions with ACHD more often underwent invasive hemodynamic monitoring with arterial lines and pulmonary artery catheters, despite similar incidences of cardiogenic shock. This is likely in part explained by the higher use of vasopressors in the ACHD admissions group and the need for invasive hemodynamics. Additionally, the presence of baseline cyanosis and organ dysfunction in patients with ACHD, particularly in Fontan patients, makes it much more challenging on physical examination and laboratory testing to accurately quantify how critically ill a patient is and may prompt a lower threshold for invasive hemodynamics.

### CICU outcomes for patients with ACHD

ACHD admissions had longer CICU and total hospital lengths of stay; however, mortality and CICU readmission rates were similar between both groups despite the substantially younger age of admissions with ACHD. With adjustment for age and sex, the odds of death remained similar for admissions with and without ACHD. Interestingly, after adjusting additionally for the greater severity of the presenting illness (using the SOFA score) among admissions with ACHD, CICU mortality was numerically lower in the ACHD group. There is likely heterogeneity within the ACHD patient population; a patient with Fontan circulation presenting in cardiogenic shock and Fontan heart failure is likely to carry a worse prognosis than a patient with an atrial septal defect presenting in atrial arrhythmia. Nevertheless, regardless of survival status, CICU presentation with more severe organ dysfunction is an important characteristic of the epidemiology of ACHD patients in the CICU. Future exploration to determine contributing causes could be important from a health care utilization and cost-effectiveness perspective. Also important to note is that even though mortality is similar between ACHD and non-ACHD admissions, the younger age of the ACHD population at the time of death represents a substantial burden of disease for this population. Even in those with ACHD who survive, it is likely that the significant comorbidity burden in this population carries a disproportionately heavy toll in younger patients who are navigating the challenges of starting families and growing careers.

As the burden of congenital heart disease mortality shifts from the pediatric to adult population, patients with ACHD will continue to have an increased burden of comorbidities, both related to their extracardiac manifestations of disease, as well as more common cardiac conditions such as coronary artery disease, diabetes, and hypertension.^1^ As this population continues to grow and age, it will be necessary for the contemporary CICU to be prepared to meet the needs of this highly comorbid and unique patient population.

### Study Limitations

There are limitations to our current study. The CCCTN registry collects observational data on only medical CICU admissions; thus, it does not evaluate ACHD patients admitted to other ICUs, including pediatric CICUs. As discussed above, our registry only records data for the first CICU admission in a given hospitalization, and thus does not inherently represent a complete analysis of a patient’s CICU resource utilization over time. Additionally, only in-hospital outcomes are collected for the CCCTN registry, and data regarding postdischarge outcomes are not collected. Data are collected by chart review by site investigators and are subject to the possibility of misclassification, particularly in the case of classifying congenital lesions. Specifically in the case of ventricular function, the CCCTN registry only captures systemic ventricular ejection fraction, not subpulmonic ventricular ejection fraction, which may be an important contributor to the underlying disease process particularly in those with increased pulmonary vascular resistance or complex lesions. Furthermore, subclassification of specific congenital lesions was only recorded for admissions after 2019 and does not account for different types of surgical repairs, limiting our ability to perform informative subgroup analysis, although most of these patients would be categorized under the “complex” category. Further subclassification among ACHD categories will be valuable in future work as subsets of types of ACHD may carry additional weight in determining prognosis. Finally, comparative data presented are subject to potential confounding and chance findings due to multiple comparisons.

## Conclusions

This epidemiological study of contemporary CICU’s in North America demonstrates that admissions with ACHD present substantially younger than their non-ACHD counterparts, have a unique set of comorbidities related to the sequelae of their congenital lesion, and utilize a distinct set of CICU resources and therapies. Our study illustrates the importance of ACHD expertise in guiding the management of critically ill ACHD patients. Further investigation into the best approach to manage specific CICU admissions, such as a cardiogenic shock and acute respiratory failure, is warranted.

PERSPECTIVES **COMPETENCY IN MEDICAL KNOWLEDGE:** Patients with ACHD admitted to the CICU require a unique subset of ICU therapies and expertise in both cardiac and noncardiac comorbidities related to their disease.

**TRANSLATIONAL OUTLOOK:** Further investigation into the best therapeutic options for patients with ACHD admitted to the CICU is needed to best serve this patient population.

## Funding support and author disclosures

Dr Morrow is a member of the TIMI Study Group, which has received institutional research grant support through Brigham and Women’s Hospital from 10.13039/100001316Abbott Laboratories, 10.13039/100020297Abiomed, 10.13039/100002429Amgen, 10.13039/100020132Anthos Therapeutics, 10.13039/100020305Arca Biopharma, 10.13039/100008207AstraZeneca, Daiichi-Sankyo, 10.13039/100014130Intarcia, 10.13039/100005565Janssen, 10.13039/100004334Merck, 10.13039/100008272Novartis, 10.13039/100004319Pfizer, Poxel, Quark Pharmaceuticals, 10.13039/100009857Regeneron, Roche, 10.13039/100004340Siemens, and Zora Biosciences; and he has received consulting fees from Abbott Laboratories, Arca Biopharma, InCarda, Inflammatix, Merck, Novartis, and Roche Diagnostics. Dr Krasuski has served as a consultant for Actelion/Janssen Pharmaceuticals, Bayer, Gore Medical, Medtronic and Neptune Medical; has received research funding from the Adult Congenital Heart Association and Actelion/Janssen Pharmaceuticals; and has served as a principal investigator for trials with Artivion, Corvia, Edwards Lifesciences, and Medtronic. Dr Newby has received research support through Duke University from 10.13039/100004374Medtronic, BioKier, and 10.13039/100016545Roche Diagnostics; and consulting honoraria from Medtronic and CSL-Behring. All other authors have reported that they have no relationships relevant to the contents of this paper to disclose.
